# Use of Postpartum Family Planning among Women Undergoing Deliveries in a Tertiary Care Hospital: A Descriptive Cross-sectional Study

**DOI:** 10.31729/jnma.7043

**Published:** 2021-11-30

**Authors:** Noora Pradhan, Anjana Dongol, Rashmi Bastakoti, Shailendra Bir Karmacharya, Om Hari Shrestha

**Affiliations:** 1 Department of Obstetrics and Gynaecology, Dhulikhel Hospital, Dhuiikhei, Kavrepalanchowk, Nepal; 2 Department of Neonatology, Paropakar Maternity and Women's Hospital, Thapathali, Kathmandu, Nepal

**Keywords:** *contraception*, *counseling*, *family planning*, *postpartum*

## Abstract

**Introduction:**

The postpartum period is a high-risk time for unintended pregnancies. A short interpregnancy interval leads to a series of complications for both the mother and the fetus. Postpartum contraceptive knowledge helps women decide the time frame for future pregnancy and prepare. The study aimed to find out the prevalence of postpartum family planning among women undergoing deliveries in a tertiary care hospital.

**Methods:**

A descriptive cross-sectional study was conducted from hospital records of all postpartum women delivering in a tertiary care hospital from Jan 2017 to Jan 2019. Ethical approval was taken from the Institutional Review Committee (IRC) of Kathmandu University School of Medical Sciences/Dhulikhel Hospital (reference number: 62/19). Convenience sampling was done. Data was entered and analyzed using Statistical Package of the Social Sciences version 26. Point estimate at 95% Confidence Interval was calculated along with frequency and proportion for binary data.

**Results:**

Out of 4205 deliveries, 1211 (28.7%) (27.33-30.06 at 95% Confidence Interval) women utilized postpartum family planning. Depot-medroxyprogesterone acetate was adopted by a majority of the participants 802 (19.1%).

**Conclusions:**

The use of postpartum contraception in this study was similar to the findings from studies done in national data and studies.

## INTRODUCTION

Postpartum Family Planning (PPFP) deals with the prevention of unintended pregnancy and closely spaced pregnancies through the first 12 months after childbirth.^1^ World Health Organization (WHO) recommends to wait for at least 18-24 months after a live birth before conceiving again to reduce the risk of adverse maternal, perinatal, and infant outcomes.^2^

PPFP has played a vital role in reducing the Maternal Mortality Ratio (MMR) in developing countries like Nepal.^3^ For which, the acceptance of family planning methods has played a vital role. However, the annual report of Department of Health Services (DoHS) 2076/77 has stated that postpartum uptake of family planning has decreased except for implant.^4^ Similarly, the Nepal Demographic Health Survey (NDHS) 2016 reported that only 13% of the women have received family planning counseling in Nepal.^5^ Proper counseling can help couples accept the family planning methods.^6^

This study aimed to find out the prevalence of postpartum family planning among women undergoing delivery during a three-year period in Dhulikhel Hospital.

## METHODS

A descriptive cross-sectional study was conducted in the Gynecology and Obstetrics Department of Dhulikhel Hospital, Kavre which provides both Basic and Comprehensive Emergency Obstetric and Neonatal Care services. The ethical consideration was taken from the Institutional Review Committee (IRC) Kathmandu University School of Medical Sciences/ Dhulikhel Hospital (KUSMS) with the approval number 62/19. All postpartum women delivered in the hospital from Jan 2017 to Jan 2019 were counseled for uptake of any family planning methods. All the postpartum women who delivered in Dhulikhel hospital irrespective of parity, mode of delivery, and postpartum women who visited Dhulikhel hospital OPD within 10 weeks of delivery were counseled on family planning. Delivered women who accepted family planning during the period were included in the study. Women with outside delivery referred for the other reason, those past 10 weeks postpartum and antenatal cases were excluded. Convenience sampling was done to select the participants in the study. The sample size of the study was calculated using the formula,

n = Z^2^ × p × q / e^2^

  = (1.96)^2^ × 0.5 × (1-0.5) / (0.04)^2^

  = 600

Where,

n= minimum required sample size
Z= 1.96 at 95% Confidence Interval (CI)
p= prevalence taken as 50% for maximum sample size
q= 1-p

Hence, the calculated sample size was 600. As we used convenience sampling, we doubled the sample size to 1200. However, we collected data of 1211 participants.

As a part of practice participants who visited during the Antenatal Care period (ANC) along with women after delivery whether primi or multi had been provided with family planning counseling and the details were recorded in the discharge summary of Dhulikhel hospital. Data were collected based on discharge paper. Assuming all forms of contraceptives measures were explained in detail to the women and the partner (whenever available) or any of the family members accompanying them, including the method, advantages, and disadvantages and finally giving them the freedom to choose based on the protocol of the hospital. The contraceptive measures offered Depot Medroxyprogesterone Acetate (DMPA), Intrauterine Contraceptive Device (IUCD), Lactational Amenorrhea Method (LAM), barrier contraception, natural family planning, and sterilization. The data was obtained for this study from the records of delivery conducted and the family planning department of Dhulikhel Hospital.

The data was entered in Microsoft Excel and descriptive statistics were obtained which included frequency and percentage. Point estimate at 95% CI was calculated along with frequency and percentage for binary data.

## RESULTS

Out of the total 4205 deliveries performed, the prevalence of post-partum contraceptive use was 1211 (28.8%) (27.33-30.06 at 95% Confidence Interval). Deliveries included all normal, vaginal (breech, twins, preterm, instrumental, vaginal birth after cesarean) deliveries and cesarean sections. Among them, 609 (50.3%) underwent delivery between January 2017 to January 2018. Similarly, 602 (49.7%) gave birth between February 2018-January 2019 (Table 1).

**Table 1:** Year-wise distribution of acceptance of family planning (n = 1211).

Time period	Acceptance of family planning n (%)
January 2017-January 2018	609 (50.3)
February 2018-January 2019	602 (49.7)

There were 2328 deliveries performed between January 2017 and January 2018 of which 609 (26.2%) accepted family planning. Similarly, of the 1877 deliveries performed between February 2018 and January 2019, 602 (32.1%) accepted family planning (Table 2).

**Table 2:** Acceptance of family planning according to the number of deliveries performed in each year.

Time period	Total number of cases	Family planning denied n (%)	Family planning accepted n (%)
January 2017-January 2018	2328	1719 (73.8)	609 (26.2)
February 2018-January 2019	1877	1275 (67.9)	602 (32.1)

Of those who accepted family planning in two years period, the majority of the participants were aged less than 25 years with mean age of 25.4±5.4 years. Considering the ethnic groups, the majority of Janajati 1292 (63.4%) accepted the family planning methods during their post-partum period (Table 3).

**Table 3:** Socio-demographic characteristics of participants accepting family planning (n=1211).

Age	n (%)
Less than 25	592 (48.9)
More than 25	619 (51.1)
Ethnicity	
Dalit	177 (14.6)
Janajati	768 (63.4)
Madhesi	6 (0.5)
Muslim	3 (0.2)
Brahmin/Chhetri	257 (21.2)

**Figure 1:**
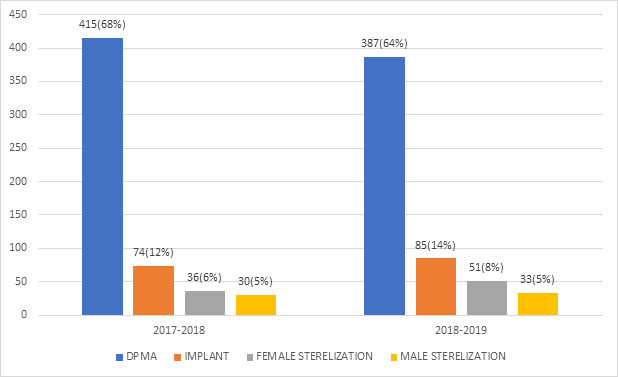
Methods of postpartum family planning adopted by the participants.

The acceptance of family planning regarding DMPA decreased from 415 (68%) to 387 (64%) from 2017-2018 to 2018-2019. There was an increase in the acceptance of implant from 74 (12%) to 85 (14%) from 2017-2018 to 2018-2019 and a similar increase in acceptance of female sterilization from 36 (6%) to 51 (8%) from 20172018 to 2018-2019. The acceptance of male sterilization was similar between two time periods. The uptake of DMPA and implant was the most accepted method. DMPA has been the most convincing and easily accepted method from the rest of all methods. However, there is no data to conclude whether they continued in the next 3 months period (Figure 1).

The population who didn't accept any method was either into LAM or wanted to carry some method after 45 days in their nearby center or had their partners working away from home which accounted for 2994 (71.3%).

## DISCUSSION

The postpartum period is potentially the ideal time to start contraception as women are strongly motivated and is within the reach of health care professionals for counseling and providing the best service needed which breaks the cascade of malnutrition and improper care of the first child along with the mother whilst other complications to the unborn.^7^

In this study, PPFP was found to be accepted by only 26.2% in 2017 and 32.1% in the year 2019 respectively. The national data from the annual report 2017/2018 depicted that PPFP uptake has been increased as compared to previous years.^4^

The study by Shrestha (2014) showed that implant was used by the majority of the participants followed by DMPA.^8^ While the current study showed the highest acceptance of DMPA, followed by implant. This might be due to the short-term acting barrier of DMPA.

Family counseling during the postpartum period might have NDHS 2016 reporting that only 13% of the women have received family planning counseling in Nepal^5^. Our study also aimed to provide family planning choices to the women and their partners after counseling to increase the uptake of PPFP. A study conducted by Goel, et al. shows that women who received advice on family planning were more likely to adopt postpartum contraception than those who were not advised at all^9^. Similar findings have been reported by studies conducted by Chabbra et al.^10^

In another study conducted in Liberia, important barriers to utilization of PPFP were lack of access and awareness of PPFP including myths and misconception.^11^ This was identified in our study as well. In our study, despite the family planning counseling most of the women denied PPFP because they either wanted to go for LAM as the exclusive breastfeeding for the six months prevents the pregnancy. A study by Wijden, has stated that LAM provides more than 98% of protection from pregnancy in the first six months postpartum.^12^ Further, in our study some participants stated that their partner was away from home due to which they do not need family planning methods currently. However, no exact information could be obtained. A study by Joshi, et al. showed that the majority of postpartum women did not use family planning methods because their husband was not at home.^13^

Though the study has significant findings, the limitation of the study includes inadequate follow-up of the patients as many of the participants were not the ones who were counseled during ANC. Further, participants hardly visit the hospital just for family planning after delivery of the child. The strength of the study is that it provided every postpartum woman structured counseling and generating awareness. Education of PPFP not only to the woman but to her partner especially during the antenatal period might provide awareness for the adoption of PPFP. The limitation is as the study is retrospective one lacks data on follow-up, thereby leaving space for future research.

## CONCLUSIONS

The use of postpartum contraception was similar to the findings from studies done in national data and studies. Among different family planning choices counseled to PP women and their husbands, DMPA has been the mostly accepted PPFP method. Both female and male sterilization rates were found to be very low. Further, most of the participants did not accept any family method options as they wanted to follow LAM. The study suggests that there is a need to promote the use of long-acting contraceptive methods such as IUCD among post-partum women as it seems to be a promising method in future years being long-acting and with no hormonal side effects. In addition to this, education on PPFP to both women and their partners during the postnatal period can play a vital role in increasing the acceptance rate of PPFP.
